# Impact of Exercise Therapy in ERAS Prehabilitation for Major Surgery: A Systematic Review

**DOI:** 10.3390/sports13090315

**Published:** 2025-09-10

**Authors:** Anna Antonia Valenzano, Paride Vasco, Gabriella D’Orsi, Tommaso Cassano, Raffaella Marzovillo, Antonio Di Stasi, Giuseppe Cibelli

**Affiliations:** 1 Department of Clinical and Experimental Medicine, University of Foggia, 71100 Foggia, Italy; anna.valenzano@unifg.it (A.A.V.); gabriella_dorsi.579495@unifg.it (G.D.); raffaella.marzovillo@unifg.it (R.M.); antonio_distasi.552940@unifg.it (A.D.S.); giuseppe.cibelli@unifg.it (G.C.); 2 Department of Humanities, University of Foggia, 71100 Foggia, Italy; 3 Department of Medical and Surgical Science, University of Foggia, 71100 Foggia, Italy; tommaso.cassano@unifg.it

**Keywords:** preoperative optimization, physical fitness, surgical recovery, nutritional support, patient engagement

## Abstract

(1) This systematic review evaluates the role of structured exercise within the Enhanced Recovery After Surgery (ERAS) protocol, a multidisciplinary approach designed to reduce surgical stress and enhance recovery. Prehabilitation, primarily through controlled physical activity, is a fundamental component of ERAS, preparing patients, both physically and mentally, for major surgery. (2) A systematic search following PRISMA guidelines was conducted using PubMed, ScienceDirect, Cochrane Library, Web of Science and Wiley databases to identify relevant studies published up to January 2025. Inclusion criteria encompassed randomized controlled trials, cohort studies, and meta-analyses reporting postoperative complications, length of hospital stay (LOS), and overall recovery. (3) A total of 64 studies met the inclusion criteria. The findings consistently demonstrate that structured exercise interventions—such as aerobic activities, resistance training, and flexibility routines—were associated with postoperative complications, shorten hospital stays, and enhance functional recovery. These interventions improve cardiorespiratory fitness, muscle strength, and psychological well-being. (4) Despite potential limitations in the systematic search, as heterogeneity of protocols, publication bias, language restrictions, the evidence supports the integration of structured exercise as a cornerstone of ERAS prehabilitation. Future research should focus on standardizing exercise protocols and exploring personalized strategies to optimize surgical outcomes across diverse patient populations.

## 1. Introduction

The use of ERAS protocols has drastically changed the paradigm of surgical care in recent years. Originally developed in the late 1990s to reduce postoperative complications and shorten hospital stays, ERAS represents a shift from traditional perioperative management to a multimodal, evidence-based approach. These protocols are built upon key principles, including preoperative optimization, minimal invasiveness, multimodal analgesia, early mobilization, and nutritional support, all aimed at attenuating the stress response to surgery and promoting faster recovery. By optimizing perioperative care and addressing both physiological and psychological elements of recovery, ERAS strategies have consistently been shown to improve patient outcomes, resulting in reduced morbidity, shorter length of stay, and enhanced patient satisfaction. Prehabilitation, which enables patients to enhance their surgical preparedness through customized therapies—particularly Adapted Physical Exercise (APE)—is a cornerstone of this approach. APE consists of individualized exercise programs designed to increase muscle strength, cardiorespiratory fitness, and overall well-being, ultimately supporting patients in coping with the physical and psychological challenges associated with surgery. According to research, APE is generally associated with a reduction in hospital stay of approximately 1.5 days and with decreases in postoperative complications of up to 30% across various surgical procedures [1]. These benefits align closely with the core objectives of ERAS protocols, which aim to optimize recovery through multimodal, evidence-based perioperative strategies. Within this context, prehabilitation—including structured APE programs—serves as a targeted approach to enhance patients’ physiological and psychological readiness for surgery. The ERAS framework has been progressively extended beyond its original application in colorectal surgery to a wide range of surgical specialties, including urological, gynecological, orthopedic, and cardiothoracic procedures [2]. Although the core principles remain consistent—such as multimodal optimization of nutrition, pain management, mobilization, and early recovery—the implementation of ERAS protocols varies considerably between specialties, resulting in heterogeneous clinical pathways [3]. This diversity highlights the need to critically synthesize the available evidence and to identify common benefits of prehabilitation interventions, particularly exercise-based strategies, in order to support the development of standardized and broadly applicable recommendations [4]. APE may exert its beneficial effects in the perioperative setting through multiple biological and physiological mechanisms. Regular structured training has been shown to modulate systemic inflammatory responses, attenuating the release of pro-inflammatory cytokines and oxidative stress, while promoting a more balanced immune function. Furthermore, exercise improves skeletal muscle mass and strength, counteracting sarcopenia and enhancing metabolic efficiency, which are critical for postoperative recovery [4]. Cardiopulmonary adaptations, including increased aerobic capacity and improved ventilatory function, further contribute to a greater functional reserve and resilience against surgical stress [3]. Collectively, these mechanisms support the rationale for integrating prehabilitation programs within ERAS pathways, as they may translate into improved tolerance to surgery and enhanced recovery trajectories.

### 1.1. The ERAS Paradigm and Its Relevance in Modern Surgery

Since its introduction in the early 2000s, ERAS has revolutionized traditional surgical care by expediting recovery and minimizing complications [5]. Recent updates from the ERAS Society in 2021 have provided comprehensive guidelines for preadmission, preoperative, intraoperative, and postoperative care, emphasizing the importance of patient education, lifestyle modifications, and tailored prehabilitation strategies.

### 1.2. The Role of Prehabilitation in Preparing Patients for Surgery

As core element of ERAS, prehabilitation optimizes patients’ physical and psychological readiness for surgery. APE is integral to this process, promoting cardiovascular fitness, muscle strength, and endurance. According to a systematic review by Hall et al. [6], patients who engaged in preoperative exercise had a 40% lower risk of postoperative complications. Furthermore, nutritional evaluations, which include diets high in protein and micronutrient supplements, are necessary to sustain energy reserves and boost immunological function, both of which are vital for the healing process following surgery. Reducing preoperative anxiety and improving mental resilience also require psychological support via mindfulness-based therapies or cognitive–behavioral therapy (CBT) [6]. These interventions improve pain management and accelerate functional recovery, aligning with ERAS goals. Prehabilitation is generally initiated in the outpatient setting during the preadmission phase, often 2–6 weeks before surgery, depending on the time available between diagnosis and the scheduled intervention. Programs are commonly delivered in hospital-based rehabilitation facilities, community centers, or as home-based supervised exercise plans, sometimes using telemedicine support to enhance accessibility. The duration of interventions varies across studies, but most range from 2 to 8 weeks, aiming to optimize functional capacity prior to surgery.

### 1.3. Scope of the Review

Despite the growing body of research, significant gaps remain in the current literature. Prehabilitation programs involving exercise vary widely in terms of type (aerobic vs. resistance), frequency, duration, and intensity, with no consensus on standardized protocols applicable across surgical contexts [4]. Furthermore, many studies suffer from methodological limitations, including small sample sizes, heterogeneous outcome measures, and inconsistent follow-up, which limit the generalizability of their findings. Some investigations report clear benefits in functional capacity and recovery, while others show less pronounced or inconclusive results, underscoring the presence of controversy within the field. These issues highlight the need for systematic synthesis of the evidence to inform future standardization of exercise-based prehabilitation strategies within ERAS pathways. In the context of the ERAS paradigm, this review will evaluate cohort studies, randomized controlled trials, and systematic reviews to give an overall view of how these interventions improve important outcomes across a range of surgical specialties, including lowering postoperative complications, reducing hospital length of stay, and improving functional recovery.

## 2. Materials and Methods

**Figure 1:**
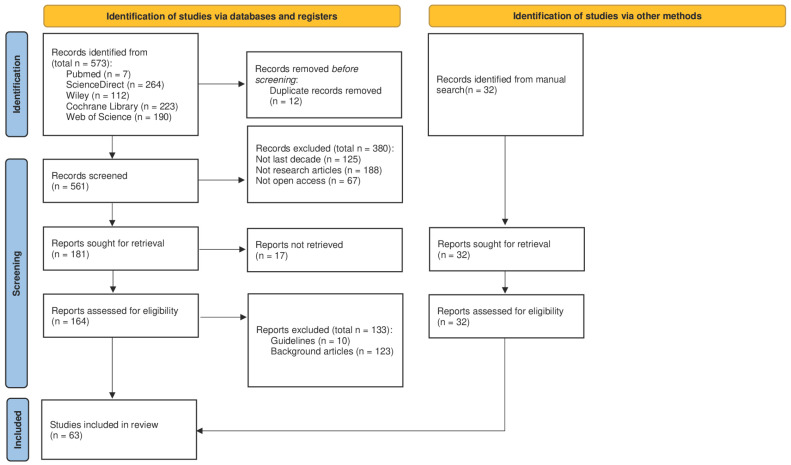
Prisma 2020 flowchart diagram for study selection. Identification of studies via databases and other methods. The arrow connect the selection activities in sequence.

This review was conducted in accordance with the PRISMA 2020 statement (Figure 1); however, no protocol was prospectively registered in PROSPERO or other registries. This represents a methodological limitation that may reduce transparency and reproducibility. The review process included a structured multi-phase screening (identification, screening, inclusion), a clearly defined search strategy, and independent reviewer assessment of studies. The systematic scoping review checklist is included as Supplementary Materials.

### 2.1. Identification Phase

The identification phase was carried out through a systematic search of the PubMed, ScienceDirect, Cochrane Library, Web of Science, and Wiley databases, covering publications from the last decade up to June 2025. This cutoff was selected to ensure the inclusion of evidence generated after the widespread implementation and consolidation of ERAS protocols in clinical practice. Restricting the search to more recent years also allowed us to capture advancements in perioperative care, exercise prescription, and nutritional support, thereby reflecting the most current scientific developments in the field. The search strategy combined predefined keywords and MeSH terms relevant to the topic, including “prehabilitation”, “structured exercise”, “Enhanced Recovery After Surgery”, “postoperative recovery”, and “surgical outcomes”. The following search string and search terms were used for the research: preoperative optimization OR enhanced recovery after surgery OR prehabilitation OR preoperative recovery AND physical exercise OR structured exercise AND nutritional support AND surgical outcomes. Filters were applied to retrieve only open access publications written in English. Studies were selected if they reported on structured exercise interventions within ERAS protocols applied to major surgeries. The initial list of references was exported and screened using the RAYYAN web-based platform, with duplicate records removed prior to analysis. No additional software was employed.

### 2.2. Screening Phase

The screening process involved a two-step evaluation of titles and abstracts conducted independently by two reviewers. Disagreements during this phase were discussed and resolved by consensus; if unresolved, a third reviewer was consulted. Eligible study designs included randomized controlled trials, cohort studies, and meta-analyses that addressed key outcomes such as postoperative complications, hospital length of stay, and global patient recovery. Articles were excluded if they were narrative reviews, clinical guidelines, conference abstracts, or book chapters. Additionally, studies published before 2015 or those not available in open access were excluded. While this criterion facilitated transparency and reproducibility, it may have limited the number of eligible studies, potentially excluding relevant evidence published behind paywalls. No restrictions were placed on surgical discipline, provided the intervention was clearly part of an ERAS pathway.

### 2.3. Inclusion Phase

Following the screening phase, full-text versions of the selected articles were retrieved and assessed for eligibility based on the predefined inclusion and exclusion criteria. Data extraction was independently performed by two reviewers using a standardized extraction form, recording variables such as authors, year of publication, country, study design, sample size, and surgical procedure. Specific attention was paid to intervention characteristics, particularly the exercise protocols, which were detailed in terms of type (e.g., aerobic, resistance, multimodal), frequency, duration, and intensity. Additional variables included nutritional support, psychological interventions, and outcomes such as length of hospital stay, postoperative complications, functional recovery, and quality of life. These variables were selected to capture the multifactorial nature of ERAS prehabilitation and to allow for meaningful comparisons across studies. Risk of bias was assessed using appropriate tools: the Cochrane RoB 2 tool for randomized controlled trials and the Newcastle–Ottawa Scale (NOS) for cohort studies. Each study was rated as having low, moderate, or high risk of bias. Effect measures were reported as risk ratios (RRs) or odds ratios (ORs) for complications, mean differences (MD) for length of stay, and MD or standardized mean differences (SMDs) for continuous outcomes related to recovery and quality of life. Due to the methodological heterogeneity of the included studies, a meta-analysis was not feasible. Instead, a narrative and thematic synthesis was conducted to summarize the role of structured exercise within ERAS protocols. Key themes and outcomes were identified by systematically comparing study designs, participant characteristics, intervention types, frequency, duration, and outcome measures. Heterogeneity was assessed by examining variations in study methodology, population characteristics, intervention components, and reported outcomes. Findings were integrated by highlighting both convergent and divergent results across studies, allowing us to achieve a comprehensive understanding of the multifactorial effects of structured exercise on postoperative recovery. This qualitative synthesis was conducted following the recommendations outlined in the PRISMA 2020 guidelines, ensuring a systematic, transparent, and reproducible approach.

## 3. Results

ERAS protocols and prehabilitation programs have demonstrated substantial benefits across various surgical contexts, significantly improving recovery outcomes and reducing complications. Across the included studies, sample sizes ranged from 30 to over 300 participants, with most trials enrolling patients undergoing major abdominal surgery, followed by thoracic and urologic procedures. The majority of participants were aged between 18 and 85 years, with most cohorts clustered in the 55–75 range, and with a balanced representation of male and female patients where reported. Intervention durations varied from 2 to 8 weeks, and follow-up periods ranged from immediate postoperative outcomes to 12 months. Given the marked heterogeneity in study designs, populations, interventions, and outcome measures, a formal meta-analysis could not be conducted. Therefore, the findings were synthesized narratively, focusing on consistent patterns across studies. Considerable heterogeneity was observed regarding surgical specialties, intervention modalities, and outcome reporting. Additionally, several studies carried risks of bias related to small sample sizes, lack of blinding, or incomplete outcome data. These limitations should be taken into account when interpreting the results. The results are presented according to key outcome domains for clarity. Table 1 provides an overview of the studies included in this review, grouped by thematic category.

### 3.1. Exercise/Physical Prehabilitation

Following video-assisted thoracoscopic lobectomy (VATS), physical activity levels declined postoperatively compared to pre-operative baselines (*p* < 0.001; *p* = 0.005; *p* = 0.027). Pain scores increased both at rest (mean difference 1.2, *p* < 0.001) and during walking (mean difference 1.4, *p* < 0.001). Fatigue, as measured by the Christensen Fatigue Scale, also worsened postoperatively (mean difference 1.7, *p* = 0.001), while sedentary activity and sleep duration remained unchanged. Functional recovery within seven days was not achieved, with fatigue (43%) and pain (33%) identified as the dominant barriers. APE interventions significantly enhanced recovery outcomes when integrated into ERAS protocols. These tailored programs improved cardiopulmonary capacity, muscle strength, and endurance. Aerobic exercise preoperatively increased VO2 max by 15% and reduced postoperative pulmonary complications by 20% in patients undergoing esophageal resection. High-intensity interval training (HIIT) reduced hospital stays by an average of two days for major abdominal surgery patients [25]. Resistance training in colorectal cancer patients improved muscle strength by 25% and functional walking capacity, contributing to better postoperative recovery and fewer complications. Multimodal prehabilitation combining aerobic and resistance training reduced surgical complications by 25% and shortened hospital stays by three days [4].

### 3.2. Nutritional Optimization

Targeted preoperative nutritional support, including protein supplementation, decreased postoperative complications by 20% and improved functional recovery. Specific interventions, such as low-AGE diets, reduced inflammatory markers and oxidative stress, supporting metabolic health during recovery. Protein-rich diets supplemented with leucine and vitamin D improved muscle strength and functional capacity, highlighting the importance of addressing nutritional deficiencies preoperatively [30].

### 3.3. Pain Management

Pain management strategies within ERAS protocols shifted toward multimodal analgesia, reducing opioid reliance by 40% while enhancing postoperative recovery [44]. Non-opioid medications such as acetaminophen, gabapentinoids, and NSAIDs effectively reduced pain, postoperative nausea and vomiting (PONV) by 30%, and restored bowel function 25% faster. The inclusion of dexmedetomidine further improved patient satisfaction and reduced pain levels [43].

### 3.4. Psychological Interventions

Psychological preparation and interventions significantly impacted patient recovery. CBT and mindfulness-based stress reduction programs reduced preoperative anxiety by up to 40%, improving adherence to recovery protocols by 25% [49]. Comprehensive preoperative education enhanced satisfaction and recovery outcomes. Mindfulness training, guided visualization, and relaxation techniques effectively managed anxiety and postoperative stress [52].

### 3.5. LOS

ERAS protocols demonstrated measurable reductions in hospital length-of-stay across surgical settings. In colorectal surgery, LOS decreased from 5.4 to 4.3 days (*p* < 0.001), and robotic liver resections reduced LOS to an average of 4.1 days compared to 5.7 days for laparoscopic approaches (*p* = 0.002) [54]. Integration of prehabilitation and ERAS strategies optimized recovery and highlighted the importance of a multidisciplinary approach.

In summary, the included studies suggest that prehabilitation and ERAS interventions are generally associated with improved postoperative outcomes, including functional recovery and reduced complications. However, the strength of evidence varies across specialties, and heterogeneity among studies warrants cautious interpretation. These findings are further contextualized in the Discussion.

## 4. Discussion

The integration of APE interventions into ERAS protocols appears to enhance surgical outcomes by supporting key physiological and psychological domains relevant to recovery. Across the included studies, exercise-based prehabilitation was generally associated with improved cardiopulmonary function, preservation of muscle mass, and accelerated postoperative recovery, while also reducing preoperative anxiety and supporting psychological resilience. When combined with nutritional and psychological interventions, these multimodal strategies underscore the holistic nature of ERAS and its potential to optimize perioperative care.

Nevertheless, the strength of evidence requires cautious interpretation. The included studies varied considerably in design, patient populations, surgical specialties, intervention modalities, and outcome measures. This heterogeneity, together with methodological limitations such as small sample sizes, lack of blinding, and incomplete reporting of follow-up, may limit the generalizability of results. Importantly, while several trials demonstrated significant improvements in outcomes such as cardiopulmonary capacity or reduced length of stay, effect sizes were often modest and context-dependent. A narrative synthesis was therefore adopted in this review, as the degree of variability precluded formal meta-analysis.

When considered by surgical specialty, APE and multimodal prehabilitation showed promising effects in major abdominal and colorectal surgery, with consistent reductions in complication rates and shorter hospital stays. In thoracic surgery, gains were primarily reflected in cardiopulmonary outcomes, whereas evidence in urological and gynecological procedures, although emerging, remains less robust. These differences highlight the importance of tailoring prehabilitation protocols to specific surgical populations and underscore the need for greater standardization in intervention design and outcome reporting.

From a practical standpoint, implementing APE in routine surgical care presents challenges, including resource availability, patient adherence, and integration within existing perioperative workflows. Variability in healthcare systems and patient populations may further influence the feasibility and scalability of such interventions. Addressing these barriers is essential to translate the benefits observed in controlled research settings into real-world practice.

This review has several limitations. First, the protocol was not prospectively registered in PROSPERO or other registries, which represents a methodological limitation that may reduce transparency and reproducibility, and potentially increase the risk of bias. Second, restricting the analysis to open access publications may have excluded some relevant studies. Third, substantial heterogeneity was observed across study designs, interventions, and outcomes, precluding a formal meta-analysis. Finally, variability in reporting of exercise intensity, adherence, and long-term follow-up limits the generalizability of the findings. These limitations should be considered when interpreting the results.

Future research should focus on large-scale, high-quality randomized controlled trials with standardized protocols, clearly defined outcomes, and longer follow-up periods to evaluate the sustainability of benefits. Studies directly comparing different exercise modalities, as well as investigating cost-effectiveness and patient-reported outcomes, would provide valuable insights. Moreover, pre-registration of review protocols should be prioritized to enhance transparency and reduce risk of bias in future systematic syntheses.

In summary, current evidence suggests that APE interventions, particularly when integrated with nutritional and psychological strategies, are generally associated with improved recovery within ERAS pathways. However, the variability and methodological limitations of the available studies necessitate cautious interpretation. Strengthening methodological rigor and addressing practical challenges will be essential for advancing the role of APE in perioperative care.

## 5. Conclusions

Prehabilitation, as defined within the ERAS framework, aims to optimize patients’ physical, nutritional, and psychological preparedness prior to surgery, and is generally associated with improved recovery outcomes. Evidence suggests that this integrated approach may contribute to fewer surgical complications, shorter recovery periods, and better overall well-being, largely through enhancing physiological fitness, supporting nutritional status, and improving psychological readiness.

Successful delivery of prehabilitation requires a coordinated, multidisciplinary effort involving surgeons, anesthesiologists, physiotherapists, dietitians, and psychologists. Standardization of exercise routines, nutritional support, and psychological interventions, alongside effective patient education, can help ensure feasibility and consistency across healthcare settings. Active patient engagement is particularly important to maximize adherence and the benefits of prehabilitation.

Future research should focus on long-term follow-up to evaluate sustained effects on recovery, recurrence, and quality of life, while also addressing variability across patient populations and surgical specialties. Investigating the biological mechanisms underpinning these benefits, as well as cost-effectiveness, will be essential to support wider implementation. Moreover, studies in implementation science are needed to identify strategies for scaling and integrating prehabilitation into routine surgical pathways.

In conclusion, current evidence supports prehabilitation as a promising strategy to enhance surgical care within ERAS protocols. However, variability among studies and methodological limitations warrant cautious interpretation, and further high-quality research is required to fully establish prehabilitation’s role in perioperative practice.

## Figures and Tables

**Table 1:** Categorization of included articles by thematic focus.

Category	Authors (Year)
Exercise/Physical Prehabilitation	de Almeida et al. (2017) [7]; Fenton et al. (2021) [8]; Ferreira et al. (2021) [9]; Fulop et al. (2021) [10]; Gillis et al. (2016) [1]; Gillis et al. (2023) [11]; Hall et al. (2023) [6]; Huang et al. (2016) [12]; Huang et al. (2022) [13]; Jack et al. (2019) [14]; Kaye et al. (2020) [15]; Koh et al. (2022) [16]; McKechnie et al. (2023) [5]; Minnella et al. (2018) [17]; Minnella et al. (2021) [18]; Mithany et al. (2023) [19]; Molenaar et al. (2023) [20]; Molenaar et al. (2023) [21]; Onerup et al. (2019) [22]; Shakya et al. (2022) [23]; Tew et al. (2018) [4]; Wang et al. (2020) [24]; West et al. (2015) [25] Wu et al. (2021) [26]; Wynter-Blyth et al. (2017) [27]; Yang et al. (2024) [28]; Yu et al. (2024) [29].
Nutritional Optimization	Bauer et al. (2015) [30]; Burden et al. (2019) [31]; Gazouli et al. (2024) [32]; Gillis et al. (2016) [1]; Gündoğu et al. (2019) [33]; Ho et al. (2024) [34]; Huang et al. (2024) [35] Khalooeifard et al. (2022) [36]; Laza-Cagigas et al. (2020) [37]; Paoli et al. (2015) [38]; Sowerbutts et al. (2024) [39]; Williams et al. (2017) [40]; Xu et al. (2022) [41].
Pain Management	Ivan et al. (2023) [42]; Kaye et al. (2020) [43]; Rawal et al. (2023) [44]; Simpson et al. (2019) [45]; Zwolinski et al. (2023) [46];
Psychological Intervention	Garland et al. (2017) [47]; Janssen et al. (2019) [48]; Levett et al. (2021) [49]; McCartney et al. (2022) [50]; Powell et al. (2023) [51]; Villa et al. (2020) [52]; Wang et al. (2022) [53].
LOS	Carli, et al., 2020 [54]; Chau, et al., 2022 [55]; Díaz-Feijoo, et al., 2022 [56]; Elfrink, et al., 2022 [57]; Gemma, et al., 2021 [58]; Gonella, et al., 2024 [59]; Hong, et al., 2023 [60]; Liu, et al., 2025 [61]; Merki-Künzli, et al., 2017 [62] Miralpeix, et al., 2019 [63].

Note: Articles are grouped based on their primary thematic focus as determined by study objectives and methodology. Some studies may conceptually overlap multiple categories but are listed under their dominant theme for clarity.

## Data Availability

No new data were created or analyzed in this study. Data sharing is not applicable to this article.

## Supplementary Materials

The following supporting information can be downloaded at: https://www.mdpi.com/article/10.3390/sports13090315/s1, Supplementary File S1: The PRISMA 2020 statement: An updated guideline for reporting systematic reviews.
